# Alleviating the consequences of nuclear disasters on views on radiation risks among physicians and patients: Fukushima experience

**DOI:** 10.7189/jogh.11.03069

**Published:** 2021-04-30

**Authors:** Tomoaki Tamaki, Akihiko Ozaki, Hisashi Sato, Masaharu Tsubokura, Yoshiyuki Suzuki

**Affiliations:** 1 Department of Radiation Oncology, Fukushima Medical University School of Medicine, Fukushima, Japan; 2 Department of Health Risk Communication, Fukushima Medical University School of Medicine, Fukushima, Japan; 3 Research Center for Community Health, Minamisoma Municipal General Hospital, Minamisoma, Fukushima, Japan; 4 Department of Breast Surgery, Jyoban Hospital of Tokiwa Foundation, Iwaki, Fukushima, Japan; 5 Department of Radiation Health Management, Fukushima Medical University School of Medicine, Fukushima, Japan

In nuclear disasters such as nuclear power plant accidents, local residents can be exposed to radioactive materials which may lead to the development of cancer in the long term. Close, uninterrupted, long-term monitoring and evaluation of affected populations is imperative, such as with the Life Span Study of the atomic bomb survivors in Hiroshima and Nagasaki [1] and the ongoing Fukushima Health Management Survey for Fukushima residents [2]. In the meantime, when cancer –disaster related or not – is newly detected in a resident, they should be able to receive appropriate care and treatment. However, there is limited evidence regarding how local oncology care could be restored or upgraded after a nuclear disaster and which stakeholders can play significant roles in this task. Unfortunately, the previous nuclear disasters such as atomic detonation in Hiroshima and Nagasaki or the nuclear accident in Chernobyl do not showcase any concrete examples on these important but under-represented issues.

In the Fukushima Daiichi Nuclear Power Plant (NPP) accident in March 2011, following the Great East-Japan Earthquake, radiation exposure to the residents around the affected area was reported to be substantially low [3]. Nevertheless, as is true with other mass casualties, the accident reportedly jeopardized the health and well-being of some vulnerable local residents, including those with cancer. For example, an increased proportion of undiagnosed cancer patients delayed their first medical consultation after realizing their symptoms during the disaster aftermath, mainly due to fluctuations in health access and social and family support [4]. However, existing evidence consists of simple observations of disaster impacts on local oncology care and lacks information about countermeasures. Hereby, in this commentary, as the radiation oncology professionals of the sole medical university in the Fukushima prefecture, we would like to introduce our experiences after the 2011 triple disaster (earthquake, tsunami, and nuclear disaster). We believe that our experiences will contribute to the existing evidence specifically in the following themes: 1) upgrading local oncology care; 2) educating physicians including other oncology health care professionals about radiation; and 3) communicating with members of the public about radiation and radiation therapy. In the following paragraphs, we extensively explain about these themes while citing our post-disaster activities until now (**Table 1**).

**Table 1:** Issues and countermeasures after the Nuclear Power Plant (NPP) accident

Issues after the NPP accident	Countermeasures which can be taken by radiation oncologists
Meeting the health needs of local residents	Ensuring the provision of comprehensive oncology care for the resident by
	• upgrading the surgical/medical/ radiation oncology services
	• enhancing network of oncology services among local centers
Inadequate/unconfident knowledge about radiation among physicians	Providing necessary information about radiation to physicians by:
	• strengthening the education on radiation in medical schools
	• providing physician colleagues about the information on effect and role of radiation in medicine
Anxiety of the general public about radiation exposure	Enhancing the knowledge and alleviating the anxiety on radiation in the general public by:
	• getting involved in the risk communication on radiation through drawing from the daily clinical experience of explaining the effect of radiation therapy to patients
	• collaborating with experts in other fields and sharing the unique experience of applying radiation to patients in daily clinical settings

## UPGRADING LOCAL ONCOLOGY CARE

**Figure:**
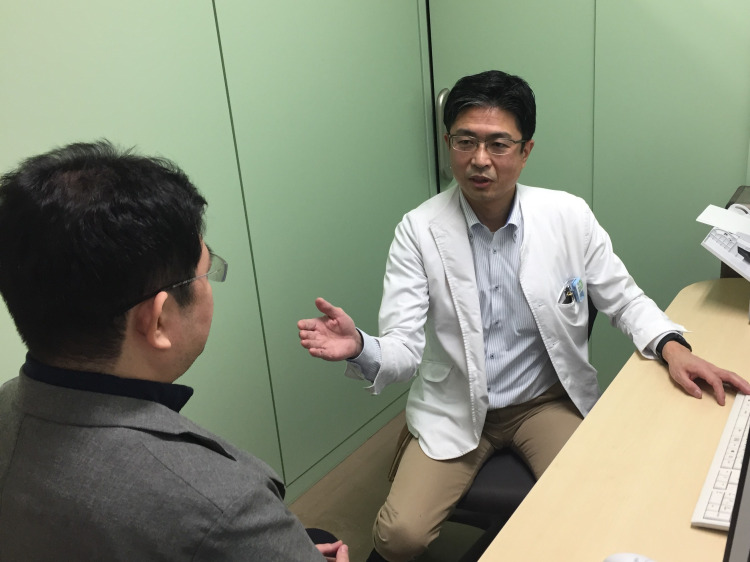
Photo: The importance of communication with patients and the public (from the authors’ own collection, used with permission).

It is of paramount importance to upgrade local oncology care in the area affected by the NPP accident. The report from United Nations Scientific Committee on the Effects of Atomic Radiation (UNSCEAR) and the International Atomic Energy Agency (IAEA) stated that the direct health effects caused by radiation exposure, including cancer, were negligible [3,5]. Nevertheless, cancer remains the most frequent cause of death in Japan [6], including in Fukushima. Furthermore, disparities in available oncology services are present, even among Fukushima prefecture areas. For example, the Soma and Futaba regions – which were significantly affected by the NPP accident –had no facilities with radiation therapy equipment for cancer patients, either before or after the accident. Since radiation therapy is a major component of cancer therapy, its absence means that Soma and Futaba residents cannot receive standard comprehensive oncology care in their hometowns. At present, when cancer patients in these regions are recommended to receive radiation therapy, they either must travel to another region or choose to receive the optimal care within available resources. Indeed, compared to the pre-disaster period, breast cancer patients experienced no apparent delays following the disaster in receiving initial treatments, surgeries, and medical therapies, as most could be provided in local facilities [7]. However, there is anecdotal evidence that some residents chose to undergo a mastectomy instead of breast-conserving surgery in order to forgo radiation therapy because of their inability to travel to distant facilities. Such stories highlight the challenges cancer patients faced in these regions due to inadequate oncology care. As clinicians and educators in the local medical university, we are determined to improve the provision of oncology services throughout the prefecture by training more radiation oncologists and providing technical support to newly established facilities. In 2017, Fukushima Medical University (FMU) formed a consortium of major hospitals in the northern part of Fukushima to provide information about radiation therapy and help build a better patient referral system. We expect to expand this effort to the entire prefecture in the near future. In 2020, FMU also formally participated in the information network of prefecture-wide hospitals, *Kibitan Net*, to facilitate providing telemedicine to Fukushima residents and support the provision of quality medical care to all residents, including rural populations.

## EDUCATING PHYSICIANS ABOUT RADIATION

Another possible contribution from radiation oncologists would be to strengthen the education university medical students receive about the biological effects of radiation and its role in medical practice. It is vital that physicians have knowledge and understanding about radiation and radiation medicine, as they were regarded by the residents inside Fukushima as the most reliable source of such information [8]. Additionally, they play a key role in helping their patients make medical decisions, including referring patients for diagnostic radiological testing and radiation therapy. However, according to a survey conducted at the FMU Hospital in 2015, about 60% of physicians reported being hesitant to prescribe computed tomography (CT) scans for their patients because of concerns and fears about radiation exposure from those radiology examinations, especially in children [9]. The hesitation is most likely expected from the heightened attention and possible public anxiety of radiation after the NPP accident. In fact, the number of scans performed in FMU is lower than in major hospitals of similar size in other prefectures (unpublished data). In order for the physicians to be able to make a sound judgement of whether CT scan or other radiation-related testing is medically justified, there is a need for educational opportunities to learn the nature and application of radiation. While we have not conducted specific surveys on radiation therapy, appropriate education on radiation and radiation therapy needs to be provided in university education, regardless of whether there has been a recent radiation accident, to make sure that the patients will not be swayed away from the necessary treatment of radiation therapy due to the excessive public fear of radiation which originates from extremely stressful events such as the NPP accident. After the 2011 NPP accident, radiation education was strengthened at FMU by the establishment of five new departments: Radiation Health Management, Radiation Life Science, Radiation Physics and Chemistry, Radiation Oncology, and Radiation Disaster Medicine. These departments complemented the existing Department of Radiation Medicine. Additionally, on a broader scope, after the NPP accident two new topics, radiation risk communication and radiation disaster medicine, were added to the section of health effects of radiation in the 2017 national core curriculum model for medical students [10]. Consequently, while the number of patients receiving radiation therapy decreased after the disaster, that number has started to increase, particularly since 2014, when the Department of Radiation Oncology was established. In fact, this number increased by 35% from 2014 to 2019.

## COMMUNICATING WITH THE PUBLIC AND OTHER EXPERTS

Lastly, an importance of communications with local residents cannot be over-exaggerated. Namely, when facing a radiation disaster that causes significant public anxiety, radiation oncologists can use their experience and knowledge to communicate with the public about radiation. The largest portion of the general public’s radiation exposure in Japan comes from medical procedures [11]. Furthermore, among the types of radiation used for medical purposes, exposure from radiation therapy accounts for largest proportion [12]. Moreover, the task of radiation oncologists is very unique in that they aim to cause a biological change in their patients—namely cancer cell death—by applying radiation. While this practice is in contrast to the other diagnostic procedures which aim to minimize a biological effect of radiation, patients who undergo high-dose radiation therapy often are either cured or receive enough symptom relief to allow them to return to their normal daily lives.

In considering messages to be conveyed to the general public from radiation oncologists, their routine communications with cancer patients provide valuable insights. When radiation oncologists communicate with their patients about radiation therapy, these patients mostly fear two things: radiation and cancer. However, because their fear of cancer is often and rightly their main concern, they hope that radiation will be a possible solution to their existing cancer. Understanding the importance of trust between cancer patients and physicians, radiation oncologists support cancer patients in their fight against cancer by communicating about the possible side effects of radiation therapy and alleviating the patients’ fears about radiation. We believe these experiences are applicable when communicating with the general public about radiation in general, even during and after a radiation disaster. Indeed, since the NPP accident, we have participated in townhall meetings and lectures with residents, schools, businesses, local governments, and governmental agencies to provide information about radiation. We also have shared our views about medical and public radiation exposure with experts around the world and the international organizations during various technical meetings.

After the NPP accident, the evaluation and management of affected residents’ health was compulsory, and we believe that radiation oncologists, along with other professions such as nuclear medicine physicians, medical physicists and radiation technologists, can contribute to this effort. First, whether caused by radiation exposure or not, since cancer remains the significant disease burden in Japan, there is a pressing need to establish a quality oncology care system within and beyond Fukushima. Further, as the experts in the field, radiation oncologists should not only contribute to radiation education for medical students and physicians but also assist radiation protection experts in the communication with the general public as knowledgeable information sources and reliable advisors in the communities regarding the medical use of radiation and unexpected radiation exposure in face of radiation disasters.

## CONCLUSION

In the event of NPP accident, even when the expected exposure of radiation is considered low, it is imperative to ensure that the comprehensive oncology care including diagnosis and treatment can be provided to the local residents during the extended period after the accident as a part of basic medical service. As professionals who apply radiation to patients in routine daily practice, radiation oncologists can contribute to the education of physician colleagues about radiation so that the physicians can provide necessary medical information to their respective patients. Simultaneously, the radiation oncologists, in collaboration with other experts, can play a valuable role in the communication with the general public about radiation and may be able to contribute to alleviating the anxiety among the public in the face of the NPP accident.
